# Alterations in choroidal circulatory dynamics and choroidal thickness before and after treatment in posterior scleritis

**DOI:** 10.1186/s12886-023-03140-8

**Published:** 2023-09-19

**Authors:** Mizuho Mitamura, Satoru Kase, Yui Yamashita, Kiriko Hirooka, Susumu Ishida

**Affiliations:** Department of Ophthalmology, Faculty of Medicine and Graduate School of Medicine, Hokkaido University, N-15, W-7, Kita-Ku, Sapporo, 060-8638 Japan

**Keywords:** Posterior scleritis, Choroiditis, Choroidal circulatory dynamics, Laser speckle flowgraphy; optical coherence tomography

## Abstract

**Background:**

Posterior scleritis is an inflammatory reaction of the sclera that occurs posterior to the ora serrata. The aim of this study was to present a case of posterior scleritis and to analyze choroidal circulatory and structural changes using laser speckle flowgraphy (LSFG) and optical coherence tomography (OCT), respectively.

**Case presentation:**

A 64-year-old man presented to our department because of hyperemia of the left eye for one week, diplopia, ocular pain, and distorted vision when looking leftward. At an initial examination, his best-corrected visual acuity was 1.0 Oculi uterque (OU), with mild conjunctival hyperemia oculus dexter (OD) and marked ciliary hyperemia oculus sinister (OS). Color fundus photographs revealed a cluster of choroidal folds extending from the macula to the inferior retinal region OS. Swept-Source OCT showed choroidal thickening OD, and bacillary layer detachment and paracentral middle maculopathy on the paracentral side of the optic nerve papilla, suggesting severe inflammation. Fluorescein angiography showed hyperfluorescence in the optic disc and window defects around the macula OU. Indocyanine green angiography showed mottled choroidal vascular hyperpermeability findings in the late stage. B-mode echography displayed thickening of the posterior wall of the left eye. Orbital magnetic resonance imaging showed the thickened posterior eyeball. The patient was diagnosed with posterior scleritis, and 30 mg of oral prednisolone was then given and tapered off over the next 4 months. The hyperemia and intraocular inflammation resolved after the treatment. The rate of change in macular blood flow assessed by the mean blur rate on LSFG was 20.5% and 20.2% decrease OD and OS, respectively, before and after treatment. The central choroidal thickness showed 8.8% and 37.8% decrease OD and OS, respectively.

**Conclusion:**

Posterior scleritis complicated with choroiditis was suggested to show different choroidal circulatory dynamics from those in other choroidal inflammations.

## Background

Posterior scleritis is an inflammatory disorder involving the sclera and episclera posterior to the ora serrata, which is likely to complicate anterior scleritis, periocular and intraocular inflammation [1]. Retino-choroidal lesions associated with scleritis include retinal striae, choroidal folds, serous retinal detachment, swollen optic disc, retinal pigment epithelial changes, and choroidal detachment [1, 2]. Clinical manifestations are very variable, depending on the exact site of inflammation, and may require differentiation from acute orbital inflammation, intraocular inflammation, Vogt–Koyanagi–Harada (VKH) disease, and ocular tumors [1–4].

To evaluate retino-choroidal lesions and assist the correct diagnosis of posterior scleritis, ophthalmic imaging is necessary including fluorescein angiography (FA), indocyanine green angiography (ICGA), and enhanced depth imaging optical coherence tomography (EDI-OCT) as well as B-mode ultrasonography. In addition, various imaging modalities including computed tomography (CT), and magnetic resonance imaging (MRI) are also employed [3, 5]. In particular, EDI-OCT, which allows noninvasive assessments of the choroidal thickness, has been useful in the evaluation of the disease state of scleritis. Hirukawa K et al. reported that central choroidal thickness (CCT) increased in acute posterior scleritis, which decreased after treatment [6]. According to other previous reports, 10 patients with 13 eyes with posterior scleritis, treated with corticosteroids (all patients) and immunosuppressive agents (7 patients), had significantly decreased mean CCT after treatment [2]. However, little is known about the relationship between morphological and circulatory changes in the choroid of posterior scleritis before and after treatment.

Laser speckle flowgraphy (LSFG) is a blood flow imaging device that uses laser scattering to noninvasively visualize the fundus circulation in two dimensions. We have employed LSFG to observe fundus circulation in various ocular fundus diseases such as optic disc melanocytoma [7], choroidal macrovessel [8], sclerochoroidal calcification [9], juxtapapillary retinal capillary hemangioblastoma [10], and choroidal lymphoma [11]. Macular mean blur rate (MBR), a relative value reflecting the choroidal blood flow velocity, increased significantly over time after systemic corticosteroid treatment together with reduction of CCT in VKH disease, a common pan-uveitis [12]. In other words, the common pattern of circulation in choroidal inflammation is a decrease in CCT and an increase in MBR before and after treatment, respectively. However, there are no reports on LSFG in posterior scleritis at all.

We herein present a case of posterior scleritis and to analyze choroidal circulatory and structural changes using LSFG and OCT, respectively.

## Case presentation

**Fig. 1:**
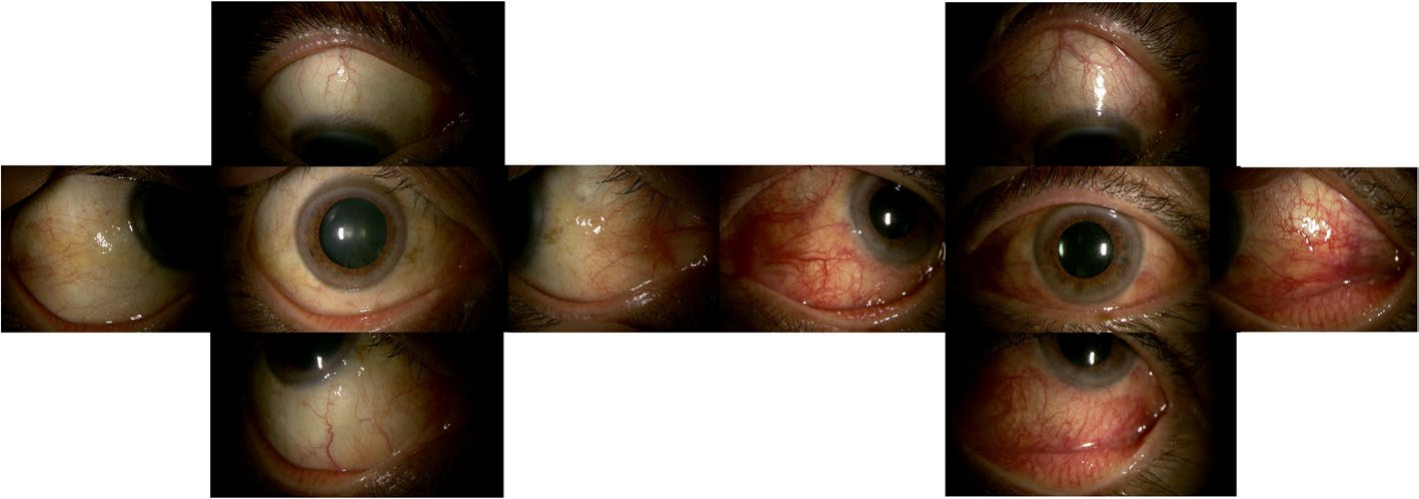
Initial findings on slit-lamp microscopy. **A**, **B** Slit-lamp microscopy detected mild conjunctival hyperemia and mild cataracts oculus dexter (OD) (**A**) and marked conjunctival and ciliary hyperemia oculus sinister (OS) (**B**)

**Fig. 2:**
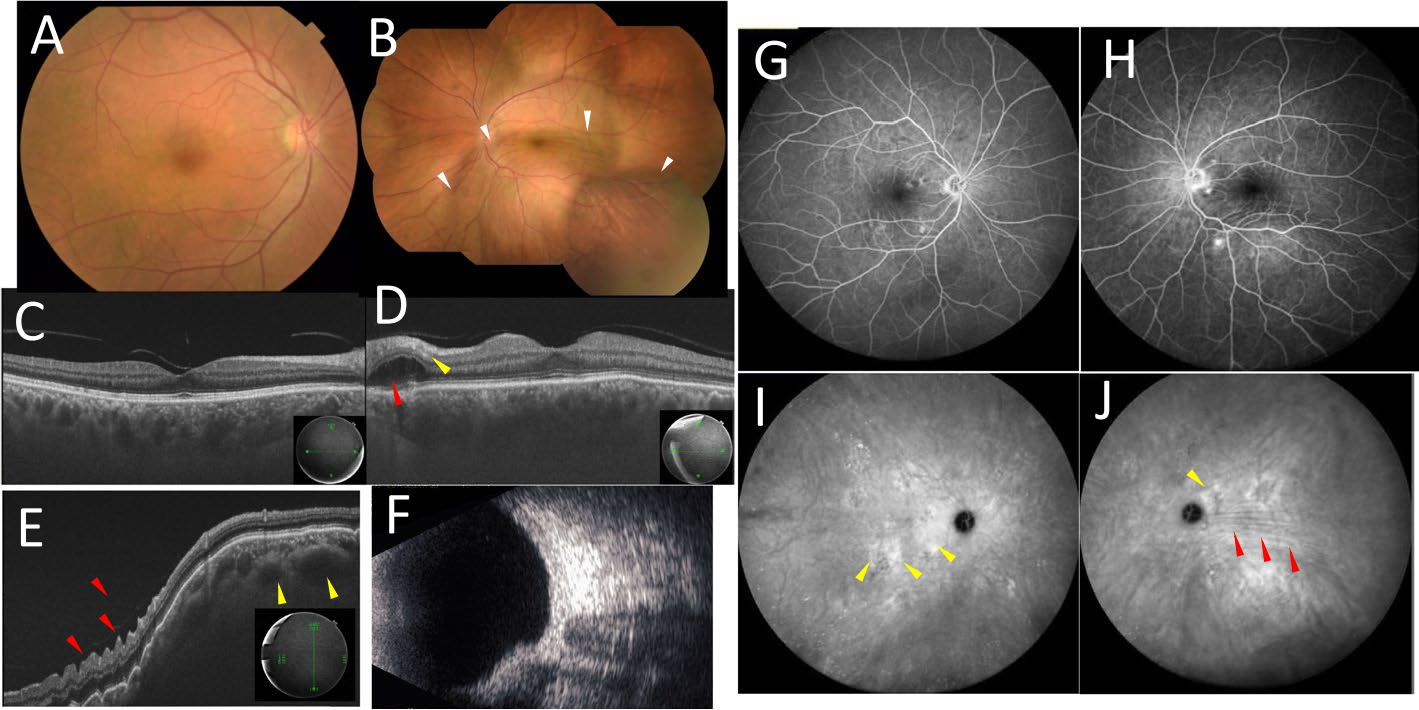
Initial findings on color fundus photography (CFP), swept-source optical coherence tomography (SS-OCT), B-mode echography, fluorescein angiography (FA), and indocyanine green angiography (ICGA) in the present case of posterior scleritis. **A**, **B** CFP revealed no abnormality OD (**A**), while a cluster of choroidal folds extended from the macula to the inferior retinal region OS (**B**, white arrowheads). **C**, **D** SS -OCT showed choroidal thickening OU, and bacillary layer detachment (**D**, red arrowhead) and paracentral middle maculopathy (**D**, yellow arrowhead) on the paracentral side of the optic nerve papilla. **E** SS-OCT of the choroidal bulge showed retinal creases (red arrowheads) and dilation of the vascular lumens (yellow arrowheads). **F** B-mode echography displayed thickening of the posterior wall of the left eye. **G**, **H** FA showed hyperfluorescence in the optic nerve papilla and window defects around the macula OU in the late stage. **I**, **J** ICGA showed mottled choroidal vascular hyperpermeability in the late stage (**I** and **J**, yellow arrowheads). Linear hyperfluorescent lesions were detected OS corresponding to the extramacular choroidal folds on CFP and SS-OCT (**J**, red arrowheads)

A 64-year-old man presented to our department because of hyperemia of the left eye for one week, diplopia, ocular pain, and distorted vision when looking leftward for one day. There were no special notes in his medical or family history. At an initial examination, his best-corrected visual acuity (BCVA) was 1.0 oculi uterque (OU), with mild conjunctival hyperemia oculus dexter (OD) (Fig. 1A) and marked ciliary hyperemia oculus sinister (OS) (Fig. 1B). Intraocular pressure was normal OU. Slit-lamp microscopy detected mild cataract OD, and pseudophakia OS. Relative afferent pupillary defect was negative, and there was limitation of eye movement of the left eye in abduction. Color fundus photographs revealed no abnormality OD (Fig. 2A), but a cluster of choroidal folds extending from the macula to the inferior retinal region OS (Fig. 1B, white arrowheads). Swept Source (SS)-OCT showed choroidal thickening OU (Fig. 2C and D ), and bacillary layer detachment (BALAD) (Fig. 2D, red arrowheads) and paracentral middle maculopathy (PAMM) (Fig. 2D, yellow arrowheads) on the paracentral side of the optic nerve papilla, suggestive of severe inflammation. SS-OCT of the choroidal bulge showed retinal creases (Fig. 2E, red arrowheads) and dilation of the choroidal vascular lumen (Fig. 2E, yellow arrowheads). B-mode echography displayed thickening of the posterior wall of the left eye (Fig. 2F). FA showed hyperfluorescence in the optic disc, and window defects around the macula OU in the late stage (Fig. 2G and H). ICGA showed mottled choroidal vascular hyperpermeability (CVH) in the late stage (Fig. 2I and J, yellow arrowheads), together with linear hyperfluorescent lesion OS corresponding to the extramacular choroidal folds (Fig. 2J, red arrowheads). Orbital MRI showed the thickened posterior eyeball. Blood tests showed no abnormalities including serum syphilis reaction, anti-toxoplasma, herpes simplex virus, herpes zoster virus, and Epstein-Barr virus antibodies, rheumatoid factor, antinuclear antibodies, C-reactive protein, and erythrocytes.

The patient was diagnosed with posterior scleritis, and 30 mg of oral prednisolone was then given and tapered off over the next 4 months. Ciliary hyperemia disappeared and the thickening of the posterior wall of the eye improved after the start of oral medication. One hundred and thirteen days after the initial visit, his BCVA was 1.0 OU, there was no hyperemia in both eyes, and the intraocular inflammation in the left eye had resolved.

The institutional review board of Hokkaido University waived ethical assessment of this clinical study because this study was a single case report with a non-invasive study. This study adhered to the tenets of the Declaration of Helsinki.

In our study, LSFG was used to evaluate changes in choroidal blood flow. Blood flow velocity was quantitatively measured by LSFG software (LSFG-NAVI, version 3.1.39.2, Softcare Ltd., Fukuoka, Japan), and relative blood flow values were obtained as the MBR in accordance with previous reports [13]. The pupils of the patient were dilated with 0.4% tropicamide (Mydrin-M; Santen Pharmaceutical Co., Ltd., Osaka) before examination, and ophthalmologic examination was performed after the light reflex in the pupils of both eyes had completely disappeared. The macular area in the LSFG image was marked manually, and the vessels were automatically segmented using threshold values defined in the system's software (LSFG Analyzer, version 3.0.47.0). A circle with a diameter of about 750 μm to the fovea was defined as the region of interest on the LSFG (Fig. 4, small circles). Four to five consecutive measurements were taken for each circle, and the mean values were used for analysis. All tests were performed by two experienced operators. Ocular perfusion pressure (OPP) was calculated using the patient's blood pressure and intraocular pressure according to previous reports [12].

There have been recent reports of detecting the choriocapillaris from en face OCT Angiography images by separating different retinal layers using a semi-automatic segmentation algorithm [14]. In this case, however, the CCT was measured manually using SS-OCT images (DRI OCT Triton; Topcon Inc., Tokyo, Japan) by two experienced examiners from the lower edge of the retinal pigment epithelium layer to the scleral border. The CCT at a first visit was defined as 800 µm because it exceeded the measurable limit of 800 µm.

**Fig. 3:**
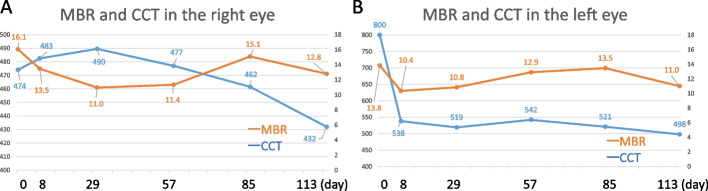
Mean blur rate (MBR) by laser speckle flowgraphy (LSFG) and the central choroidal thickness (CCT) in the present case of posterior scleritis. **A** The MBR decreased temporarily at acute phase but gradually increased at subacute phase. The CCT decreased during the recovery phase. **B** The MBR decreased temporarily at acute phase but gradually increased at subacute phase. The CCT decreased immediately after the start of treatment

The MBR values OD are shown in Fig. 3A as follows: 16.1, 13.5, 11.0, 11.4, 15.1, and 12.8 at the initial visit, 8 (acute phase), 29, 57, 85 (recovery phase), and 113 days after the initial visit, respectively. The MBR values OS are shown in Fig. 3B as follows: 13.8, 10.4, 10.8, 12.9, 13.5, and 11.0 at the initial visit, 8, 29, 57, 85, and 113 days after the initial visit, respectively. The rate of change in macular blood flow assessed by the MBR on LSFG was 20.5% and 20.2% decrease OD and OS, respectively. The both eyes decreased temporarily but gradually increased (and finally slightly decreased). OPP was 61.7, 68.1, 50.6, 48.3, 58.5, and 54.2 mmHg OD and 61.7, 68.7, 52.2, 49.2, 61.2, and 55.2 mmHg OS at the initial visit, 8, 29, 57, 85, and 113 days after the initial visit, respectively, revealing no significant changes in both eyes.

The CCT values OD were 474, 483, 490, 477, 462, and 432 µm at the initial visit, 8, 29, 57, 85, and 113 days after the initial visit, respectively (Fig. 3A). The CCT values OS were 800, 538, 519, 542, 521, and 498 µm at the initial visit, 8, 29, 57, 85, and 113 days after the initial visit, respectively (Fig. 3B). The rate change of CCT showed 8.8% and 37.8% decrease OD and OS, respectively. The CCT OD increased temporarily after the start of treatment, and then decreased slightly thereafter. The CCT OS decreased immediately after the start of treatment.

## Discussion and conclusion

The present study demonstrated novel findings on multimodal imaging techniques in posterior scleritis complicated with choroiditis, so as to better understand clinical manifestations of the disease. In our case, the MBR decreased temporarily at acute phase but gradually increased at subacute phase and finally slightly decreased at recovery phase. The CCT was thickened before treatment, which gradually decreased after treatment.

Lane J et al. reported that posterior scleritis presented with retinal detachment (13%), macular edema (20%), choroidal involvement (22%), and uveal effusion (19%) [15]. Liu et al. compared the OCT findings between patients with VKH disease and posterior scleritis and demonstrated that the frequency of subretinal structures was higher in acute VKH disease patients (80%) compared to those in patients with posterior scleritis (15%). However, the frequency of RPE folds was higher in posterior scleritis (62%) than in acute VKH disease (55%) [16]. In our case, BALAD on OCT suggested strong inflammation as is often seen in VKH disease. PAMM suggested retinal ischemia in the middle layer. Papillary hyperfluorescence on FA suggested inflammation spread to retinal vessels as well. Choroidal folds were also present in our case. These ophthalmic findings are consistent with common findings observed in posterior scleritis with strong inflammation.

The current case clearly depicted CCT alterations before and after corticosteroid administration. Histopathologic examination of seven cases with posterior scleritis demonstrated that the choroid was involved in all enucleated eyes and was thickened with inflammatory infiltrates and choroidal vasculitis [1]. The report of increased CCT in acute posterior scleritis and decreased CCT after treatment [2, 6] was supported by the histology-proven choroidal thickening due to inflammation. The SS-OCT findings in the present case were consistent with previous reports, and the OCT findings are considered to reflect choroidal inflammation verified in histological observations.

ICGA has demonstrated diffuse zonal hyperfluorescence in the choroid of posterior scleritis, indicating CVH in this disease state [17]. In our case, ICGA showed mottled CVH in the late phase and there was dilation of the choroidal lumen on OCT in the highly inflamed area. The CVH could be caused by scleral inflammation-mediated strangulation of scleral branches from the short and long posterior ciliary arteries together with imbalanced blood flow entry to choroid, and/or be associated with choroidal vasculitis seen in histopathologic examination [1].

**Fig. 4:**
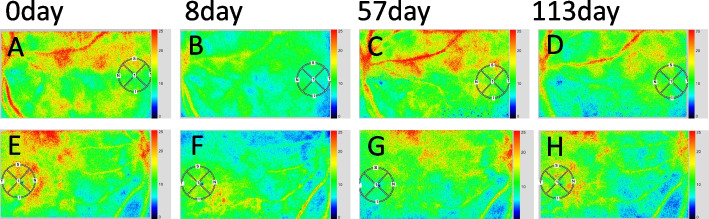
LSFG color map in the present case of posterior scleritis. **A**, **B**, **C**, **D** LSFG color map in the left eye showed a vertical difference because of the inferior choroidal bulge caused by the nodular lesion of scleritis. **E**, **F**, **G**, **H** LSFG color map in the right eye showed no vertical difference

To our knowledge, this was the first report to show LSFG on posterior scleritis complicated with choroiditis. In our case of posterior scleritis, increased MBR reflects high choroidal bloodstreams before treatment, which was consistent with the findings of CVH on ICGA; however, posterior scleritis in our case showed different circulatory dynamics from those in common choroiditis such as VKH disease, in which MBR decreased before treatment. The transition of MBR in this case was thought to depend on the reciprocal degree of suppression of scleritis and choroiditis. In contrast, the CCT consistently descended. When scleral inflammation was the predominant cause before treatment, choroidal blood flow was hyperperfused in response to strangulation of the posterior ciliary arteries in the sclera. In addition, LSFG of the left eye showed that the inferior choroidal bulge caused by the nodular lesion of scleritis reduced choroidal blood flow by mechanical compression, resulting in a vertical difference in the LSFG color map (Fig. 4). The similar findings have been reported in our previous reports regarding other ocular conditions such as sclerochoroidal calcification and focal scleral nodule [9, 18]. In the acute phase (0 ~ 8 days), the scleral inflammation initially resolved to a longer extent with the start of corticosteroid treatment, while the choroidal inflammation persisted to a certain extent. At this time, the choroidal blood flow decreased concurrently with the reduction of CVH due to the improvement of posterior scleritis in the relative balance of blood flow. During the subacute and recovery phase (29 ~ 85 days), while scleral inflammation was further reduced, choroidal inflammation was more predominantly reduced, resulting in a gradual increase in MBR as a usual common recovery-phase of choroiditis.

There are limitations on LSFG in this study. First, a circle with a diameter of about 750 μm to the fovea was defined as the region of interest on the LSFG, but LSFG color map in the left eye showed a vertical difference because of the inferior choroidal bulge caused by the nodular lesion of scleritis, which may affect acquisition of the MBR value. Second, the blood flow velocity on LSFG cannot be evaluated separately for retinal arteries and veins, so choroidal blood flows should be analyzed using multimodal imaging including ICGA.

In conclusion, posterior scleritis complicated with choroiditis was suggested to show unique choroidal circulatory dynamics characterized by a bimodal pattern.

## Data Availability

N/A

## Abbreviations

LSFG: Laser speckle flowgraphy
OCT: Optical coherence tomography
BCVA: Best-corrected visual acuity
OU: Oculi uterque
OD: Oculus dexter
OS: Oculus sinister
SS: Swept-source
FA: Fluorescein angiography
ICGA: Indocyanine green angiography
VKH disease: Vogt–Koyanagi–Harada disease
MRI: Magnetic resonance imaging
EDI-OCT: Enhanced depth imaging optical coherence tomography
CCT: Central retinal thickness
BALAD: Bacillary layer detachment
PAMM: Paracentral middle maculopathy
CVH: Choroidal vascular permeability
MBR: Mean blur rate
OPP: Ocular perfusion pressure
